# *Helicobacter pylori* Seropositivity and Risk of Lung Cancer

**DOI:** 10.1371/journal.pone.0032106

**Published:** 2012-02-24

**Authors:** Jill Koshiol, Roberto Flores, Tram K. Lam, Philip R. Taylor, Stephanie J. Weinstein, Jarmo Virtamo, Demetrius Albanes, Guillermo Perez-Perez, Neil E. Caporaso, Martin J. Blaser

**Affiliations:** 1 Division of Cancer Epidemiology and Genetics, National Cancer Institute, National Institutes of Health, U.S. Department of Health and Human Services, Bethesda, Maryland, United States of America; 2 Department of Chronic Disease Prevention, National Institute for Health and Welfare, Helsinki, Finland; 3 Departments of Medicine and Microbiology, New York University School of Medicine, New York, New York, United States of America; National Cancer Center, Japan

## Abstract

Lung cancer is the leading cause of cancer mortality worldwide. *Helicobacter pylori* (*H. pylori*) is a risk factor for distal stomach cancer, and a few small studies have suggested that *H. pylori* may be a potential risk factor for lung cancer. To test this hypothesis, we conducted a study of 350 lung adenocarcinoma cases, 350 squamous cell carcinoma cases, and 700 controls nested within the Alpha-Tocopherol, Beta-Carotene Cancer Prevention Study (ATBC) cohort of male Finnish smokers. Controls were one-to-one matched by age and date of baseline serum draw. Using enzyme-linked immunosorbent assays to detect immunoglobulin G antibodies against *H. pylori* whole-cell and cytotoxin-associated gene (CagA) antigens, we calculated odds ratios (ORs) and 95% confidence intervals (95% CIs) for associations between *H. pylori* seropositivity and lung cancer risk using conditional logistic regression. *H. pylori* seropositivity was detected in 79.7% of cases and 78.5% of controls. After adjusting for pack-years and cigarettes smoked per day, *H. pylori* seropositivity was not associated with either adenocarcinoma (OR: 1.1, 95% CI: 0.75–1.6) or squamous cell carcinoma (OR: 1.1, 95% CI: 0.77–1.7). Results were similar for CagA-negative and CagA-positive *H. pylori* seropositivity. Despite earlier small studies suggesting that *H. pylori* may contribute to lung carcinogenesis, *H. pylori* seropositivity does not appear to be associated with lung cancer.

## Introduction

Lung cancer kills more people worldwide (over 1 million each year) than any other cancer [1]. Although smoking is the primary cause, most smokers (≥80%) never develop lung cancer [2], suggesting that oncogenesis requires additional co-factors. Infections and immune responses that mediate inflammation may contribute to lung carcinogenesis [3], [4]. Evidence supporting this hypothesis includes associations of lung cancer with 1) elevated inflammatory markers, such as C-reactive protein, interleukin (IL)-6, and IL-8 [5], [6]; 2) chronic obstructive pulmonary disease, to which infections can contribute [7], [8]; 3) human leukocyte antigen polymorphisms in genome-wide association studies [9], [10]; and 4) overt infections like tuberculosis and pneumonia [3], [7]. In addition, Jaagsiekte sheep retrovirus causes ovine pulmonary adenocarcinoma (OPA), a malignancy occurring in sheep [4]. OPA has similar histology to human lung adenocarcinoma, bronchiolar-alveolar adenocarcinoma in particular. In humans, lung adenocarcinoma occurs at younger ages more often than squamous cell carcinoma [4], which is consistent with an infectious origin since some infection-related cancers occur at younger ages [11], [12].

One microbe postulated to play a role in lung cancer is *Helicobacter pylori* (*H. pylori*) [13], [14]. *H. pylori* is a key etiologic agent in the development of distal stomach cancer [15]. A gram-negative spiral-shaped bacterium, *H. pylori* colonizes the gastric mucosa, inducing local inflammation and a systemic immune response [16]. *H. pylori* has been classified as a group 1 carcinogen for stomach cancer by the International Agency for Research on Cancer [17]. *H. pylori* can be broadly categorized into two groups: type I strains, which express the cytotoxin-associated gene (*cagA*), and type II strains, which do not [18]. *CagA*-positive strains affect gastric epithelial cell signal transduction, which can affect the cytoskeleton, inflammatory cascades, and mitogenic pathways [16], [18].

*H. pylori* could potentially affect the lungs in several ways. Lipopolysaccharide is the major component of the cell wall of gram-negative bacteria like *H. pylori*. Lipopolysaccharide stimulates the production of pro-inflammatory cytokines including IL-1, IL-6, and tumor necrosis factor (TNF)-alpha. This local inflammation can have systemic effects [16], [19], [20]. *H. pylori* persistence leads to chronic inflammation and immune stimulation, which could contribute to carcinogenesis or conditions associated with lung cancer, such as chronic bronchitis [16]. The lungs develop embryologically from the same endodermal cells that line the gastrointestinal (GI) tract and contain cells that produce peptide hormones like gastrin [14]. Therefore, higher plasma levels of gastrin due to *H. pylori* in the stomach might promote cellular proliferation in the lungs as well [14].

It is also possible that gastric *H. pylori* colonization could decrease the risk of lung cancer. *H. pylori* prevalence has declined over the last 70 years, accompanied by a marked decrease in noncardia gastric cancer and an increase in esophageal adenocarcinoma [21]. Similar to the esophagus where the proportion of cancers due to adenocarcinomas are increasing in relation to squamous cell cancers, the relative proportion of lung adenocarcinoma has been increasing [22]. With the observed inverse association between *H. pylori* and esophageal adenocarcinoma in Western countries and the lack of association with esophageal squamous cell carcinoma, an inverse association between *H. pylori* and lung adenocarcinoma but no association with lung squamous cell carcinoma also could be hypothesized.

Prior assessment of the association between *H. pylori* and lung cancer has been limited, with fewer than 75 cases in each of five case-control studies [13], [14], [23], [24], [25]. A recent meta-analysis including four of these studies calculated a pooled odds ratio (OR) of 3.2 [95% confidence interval (CI): 1.1–9.5], but the authors noted marked heterogeneity in the results from these studies [26]. Using existing *H. pylori* seropositivity data from previous studies in the Alpha-Tocopherol, Beta-Carotene Cancer Prevention Study (ATBC) cohort of male Finnish smokers [27], [28], [29], [30], we found suggestive evidence of an inverse association of *H. pylori* with lung adenocarcinoma, but not squamous cell carcinoma (see Methods section). Based on these intriguing preliminary data and the lack of well-powered, high-quality studies, we designed the first prospective study of *H. pylori* seropositivity and lung cancer in ATBC.

## Materials and Methods

### Study population

The ATBC Study, a randomized, double-blind, placebo-controlled, primary prevention trial, was designed to test the hypothesis that daily supplementation with alpha-tocopherol, beta-carotene, or both would reduce the incidence of lung or other cancers among male smokers. Details of this study have been published [31]. In brief, Finnish males between 50 and 69 years of age were identified through the Central Population Register. Questionnaires were mailed to those with available addresses. Men who smoked ≥5 cigarettes per day and who agreed to participate were mailed an invitation to a local field station for further evaluation. After excluding men with proven malignancy (other than nonmelanoma skin cancer or carcinoma in situ), severe angina on exertion, chronic renal insufficiency, liver cirrhosis, chronic alcoholism, current anticoagulant therapy, other medical problems that could limit participation for 6 years, or current use of supplements with vitamin E (>20 mg/d) or vitamin A (>20,000 IU/d = 2000 retinol equivalents) or beta-carotene (>6 mg/d), 29,246 men were randomized. Later, 113 men were found to be ineligible, leaving 29,133 eligible Finnish male smokers enrolled in ATBC between 1985 and 1988. Both the US National Cancer Institute and the Finnish National Public Health Institute institutional review boards approved the ATBC Study, and all participants provided written informed consent. The men continue to be followed as a cohort since the trial ended in 1993.

### Preliminary evaluation using existing data

We used existing *H. pylori* seropositivity data from previous studies in ATBC [27], [28], [29], [30] to conduct a preliminary evaluation of the association between *H. pylori* and lung cancer. Enzyme-linked immunosorbent assay (ELISA) measurements of *H. pylori* seropositivity were available from 98 lung cancer cases (11 with documented adenocarcinoma and 33 with squamous cell carcinoma) and over 1500 subjects without lung cancer. The preliminary data revealed no apparent association between *H. pylori* seropositivity and lung cancer overall (OR: 0.96, 95% CI: 0.61–1.5). However, we observed a trend toward an inverse association with adenocarcinoma (OR: 0.46, 95% CI: 0.14–1.5), which was stronger for CagA-positive *H. pylori* strains (OR: 0.38, 95% CI: 0.07–2.0).

### Case and control definition

**Figure 1:**
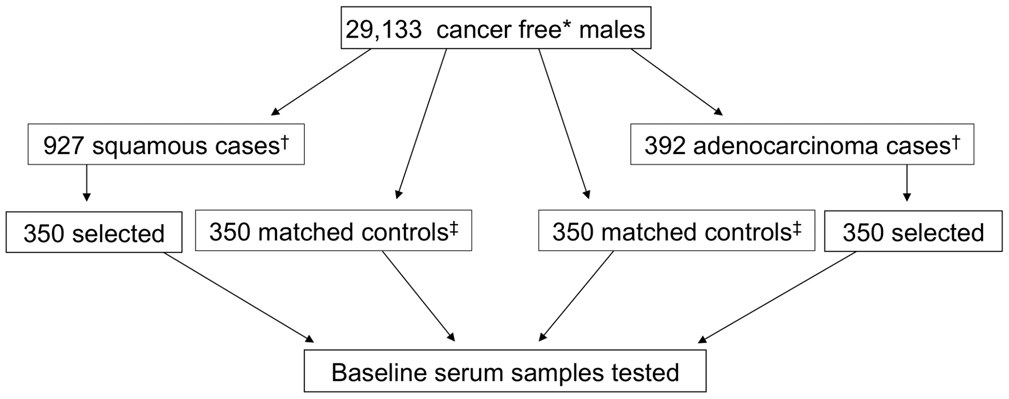
Lung cancer case and control selection for *Helicobacter pylori* serology testing within the ATBC cohort. *Individuals with nonmelanoma skin cancer were not excluded. †Eligible cases included all first primary lung squamous cell carcinoma or adenocarcinoma cases diagnosed at least 1 year after the baseline draw date through 30 July 2007. ‡Controls were matched one-to-one by age at baseline serum draw (+/−5 years) and date of baseline serum draw (+/−30 days). Controls had to be alive and cancer free at the date that the corresponding case was diagnosed.

Lung cancer cases were identified using the International Classification of Diseases, 9^th^ Revision (ICD-9) [32] code 162, through the Finnish Cancer Registry, which provides nearly 100% coverage of all cancer cases in Finland [33]. Histology was determined using the International Classification of Diseases for Oncology, 2^nd^ edition (ICD-O-2), and 3^rd^ edition (ICD-O-3). Squamous cell carcinoma included malignant ICD-O codes of 8070. Adenocarcinoma included malignant ICD-O codes of 8140 (adenocarcinoma), 8250 (bronchioloalveolar carcinoma), and 8260 (papillary carcinoma). Cases were selected from among all first primary lung squamous cell carcinoma or adenocarcinoma cases diagnosed at least 1 year after the baseline draw date through 30 July 2007. We randomly selected 350 from 927 eligible squamous cell carcinoma cases and 350 from 392 eligible adenocarcinoma cases (Figure 1). Controls were matched one-to-one by age at baseline serum draw (+/−5 years) and date of baseline serum draw (+/−30 days). Controls had to be alive and cancer free at the date that the corresponding case was diagnosed.

### *Helicobacter pylori* testing

We tested fasting serum specimens collected at baseline and stored in aliquots at −70°C for immunoglobulin G antibodies against *H. pylori* whole-cell and CagA antigens using ELISAs [34], [35]. Eleven samples (four from cases and seven from controls) lacked sufficient serum for testing. We calculated optical density ratios relative to co-analyzed laboratory standards and classified optical density ratios ≥1.0 for the whole-cell antigen assay and ≥0.35 for the CagA antigen assay as positive. We evaluated *H. pylori* seropositivity in two ways. First, we examined any *H. pylori* seropositivity (whole-cell or CagA seropositivity) compared to seronegativity for both whole-cell and CagA antigens. Second, we split *H. pylori* seropositivity into whole-cell and CagA seropositivity. Twelve individuals were classified as seropositive for CagA and seronegative for antibodies against the whole-cell antigen. A previous study comparing serology with gastric biopsy culture found that individuals who are negative for *H. pylori* whole-cell antibodies but positive for CagA antibodies had CagA-positive *H. pylori* organisms present in their stomach by culture [36]. Therefore, we classified individuals who were seronegative for both whole-cell and CagA antibodies as *H. pylori* negative, individuals seropositive for whole-cell but seronegative for CagA as *H. pylori*-seropositive/CagA seronegative (CagA-negative strains), and individuals seropositive for CagA, regardless of whole-cell serostatus, as CagA seropositive (CagA-positive strains).

In addition to the 1389 samples from lung cancer cases and controls, 3 quality control serum samples aliquoted from a single large pool were equally distributed among 37 different batches. All samples were assayed in duplicate in the same batch by experienced technicians blinded to case-control status. In the case of indeterminate results (i.e., if the values from the duplicate sample straddled the seropositivity threshold), a repeat analysis was conducted on the residual of the original aliquot and averaged across all results, excluding obvious outliers. 95.9% of the samples were resolved with two runs in the whole-cell ELISA, and 94.9% of the samples were resolved with two runs in the CagA ELISA. More than 99% of samples were resolved with two or three runs (99.5% for whole-cell and 99.4% for CagA). The remainders were resolved with four runs.

### Statistical analysis

Based on the observed prevalence of *H. pylori* seropositivity in a prior ATBC study with a prevalence of 45% among controls [27], we designed this study to have 90% power to detect ORs of 0.70 or 1.42 for CagA and all lung cancers combined. Assuming an overall *H. pylori* exposure prevalence of 75% among the controls, the minimal detectable ORs at 90% power were 0.68 or 1.53 for all lung cases compared to controls. We calculated within-batch coefficients of variation (CV) for the whole-cell and CagA antigen assays based on continuous optical density values from the 111 pooled QC samples (three within each batch). The within batch CVs were 12.9% for the whole-cell assay and 25.5% for the CagA assay, similar to the CVs of 15% and 20%, respectively, in a previous study of *H. pylori* and gastric cancer in ATBC using the same laboratory [27].

We used conditional logistic regression to calculate separate ORs and 95% CIs for the association of lung adenocarcinoma and squamous cell carcinoma with *H. pylori* seropositivity, using the categories described above. Results for adenocarcinoma and squamous cell carcinoma were similar according to the Wald test for homogeneity [37] (P = 0.8), so we also evaluated all lung cancer patients combined.

Potential confounders included ATBC treatment group (placebo, alpha-tocopherol, beta-carotene, or both), age at randomization (continuous), baseline pack-years (continuous), baseline number of cigarettes per day (continuous), tooth loss (continuous), dentures (yes/no), obesity (ordinal body mass index <25, 25–30, 30+), asthma (yes/no), emphysema (yes/no), bronchitis (yes/no), and education (<elementary, elementary, partial junior high, junior high, senior high, graduate). We evaluated the importance of these variables by removing each covariate from the model one by one through backwards modeling and determining whether the ORs for CagA-negative strains and CagA-positive strains changed by at least 10% in comparison with the full model. No covariate met this criterion. However, we included pack-years and smoking amount (cigarettes per day) in all final models to adjust for smoking exposure, in accord with several recent studies [8], [38], [39], [40]. Adjusting for pack-years or amount of smoking had little effect on the ORs (e.g., 2–3% for any *H. pylori* seropositivity).

#### Sensitivity analyses

A previous study of *H. pylori* seropositivity in Finland found that antibody levels remain relatively stable over time in this population [41], and in ATBC the association between *H. pylori* and gastric cancer was similar when stratified by time to diagnosis [27]. However, to evaluate the effect of potential changes in *H. pylori* antibody levels over time, we stratified by median time since blood draw. Since microbe-related cancers often occur at younger ages than those not related to infection [11], [12], we also stratified by median age at diagnosis and evaluated whether the association between any *H. pylori* seropositivity and lung cancer varied by age at diagnosis (i.e., whether there were departures from multiplicative joint effects) using the likelihood ratio test (LRT) for interaction terms [37], [42].

## Results

The case and control series were similar with regard to age, median cigarettes per day, and other characteristics (Table 1). As expected based on the primary results of the trial [43], [44], squamous cell cases were slightly less likely to have received placebo and slightly more likely to have received beta-carotene than controls. Cases had a higher number of pack-years than controls, but adjusting for pack-years did not affect the association between *H. pylori* and lung cancer, as described in the Methods section. Among the 696 cases and 693 controls with sufficient sample available, 555 cases (79.7%) and 544 controls (78.5%) had antibodies against either *H. pylori* whole-cell or CagA antigens (Table 2). Thirty-seven percent of cases (N = 258) and 34.3% of controls (N = 238) were seropositive for CagA-negative strains of *H. pylori*, and 42.7% of cases (N = 297) and 44.2% of controls (N = 306) were seropositive for CagA-positive strains. Of the 603 CagA-positive individuals, 591 [289 out of 297 cases (97.3%) and 302 out of 306 controls (98.7%)] also were positive for antibodies against the whole-cell antigens.

**Table 1:** Characteristics of randomly selected lung adenocarcinoma and squamous cell carcinoma cases and matched cancer-free controls from the Alpha-Tocopherol, Beta-Carotene Cancer Prevention Study.

Characteristic	Subgroup	Adenocarcinoma		Squamous cell carcinoma	
		Cases	Controls*	Cases	Controls*
Median age at baseline serum draw		58.0	58.0	59.8	59.6
Median date of baseline serum draw		1/23/1987	1/23/1987	1/22/1987	1/23/1987
Treatment group, N (%)					
	Placebo	84 (24.0)	94 (26.9)	75 (21.4)	97 (27.7)
	Beta-carotene	88 (25.1)	85 (24.3)	110 (31.4)	87 (24.9)
	Alpha-tocopherol	89 (25.4)	83 (23.7)	80 (22.9)	86 (24.6)
	Alpha-tocopherol+beta-carotene	89 (25.4)	88 (25.1)	80 (22.9)	85 (24.3)
Education level, N (%)					
	Primary school or lower	282 (80.6)	275 (78.6)	291 (83.1)	288 (82.3)
	High school or higher	68 (19.4)	75 (21.4)	59 (16.9)	62 (17.7)
Obesity, N (%)					
	BMI† <25	178 (50.9)	136 (38.9)	157 (44.9)	129 (36.9)
	BMI† 25–<30	144 (41.1)	160 (45.7)	141 (40.3)	165 (47.1)
	BMI† ≥30	28 (8.0)	54 (15.4)	52 (14.9)	56 (16.0)
Median cigarettes per day (range)		20 (5–55)	20 (5–75)	20 (5–60)	20 (5–60)
Median pack-years (range)		42 (1–121)	35 (1–101)	42 (4–111)	35 (1–123)
Asthma, N (%)		14 (4.0)	8 (2.3)	19 (5.4)	7 (2.0)
Emphysema, N (%)		32 (9.1)	24 (6.9)	36 (10.3)	21 (6.0)
Bronchitis, N (%)		41 (11.7)	27 (7.7)	47 (13.4)	25 (7.1)
Median number of lost teeth (range)		4 (1–5)	4 (1–5)	4 (2–5)	4 (1–5)
Dentures, N (%)		238 (68.0)	216 (61.9)	261 (74.8)	241 (68.9)

*Controls matched with cases on age at baseline serum draw and date of baseline serum draw.

† BMI = body mass index.

**Table 2:** Association of *Helicobacter pylori* (*H. pylori*) seropositivity with risk of lung adenocarcinoma and squamous cell carcinoma in the Alpha-Tocopherol, Beta-Carotene Cancer Prevention Study.

Model	*H. pylori* serostatus	Histology	Cases, N* (%)	Controls, N* (%)	OR (95% CI)†
1	*H. pylori* seronegative	Adenocarcinoma	74 (21.2)	79 (22.8)	1.0 (referent)
	*H. pylori* seropositive		275 (78.8)	268 (77.2)	1.1 (0.75–1.6)
2‡	*H. pylori* seronegative		74 (21.2)	79 (22.8)	1.0 (referent)
	CagA-seronegative‡		125 (35.8)	119 (34.3)	1.1 (0.71–1.7)
	CagA-seropositive‡		150 (43.0)	149 (42.9)	1.1 (0.73–1.7)
1	*H. pylori* seronegative	Squamous cell carcinoma	67 (19.3)	70 (20.2)	1.0 (referent)
	*H. pylori* seropositive		280 (80.7)	276 (79.8)	1.1 (0.77–1.7)
2‡	*H. pylori* seronegative		67 (19.3)	70 (20.2)	1.0 (referent)
	CagA-seronegative ‡		133 (38.3)	119 (34.4)	1.3 (0.82–1.9)
	CagA-seropositive ‡		147 (42.4)	157 (45.4)	1.0 (0.65–1.6)
1	*H. pylori* seronegative	All lung cancer	141 (20.3)	149 (21.5)	1.0 (referent)
	*H. pylori* seropositive		555 (79.7)	544 (78.5)	1.1 (0.86–1.5)
2‡	*H. pylori* seronegative		141 (20.3)	149 (21.5)	1.0 (referent)
	CagA-seronegative ‡		258 (37.1)	238 (34.3)	1.2 (0.87–1.6)
	CagA-seropositive ‡		297 (42.7)	306 (44.2)	1.1 (0.79–1.5)

*N's do not sum to total due to missing values.

† Odds ratios (ORs) and 95% confidence intervals (CIs) were adjusted for baseline pack-years and total number of cigarettes per day.

‡ Single model for CagA-seropositive and CagA-seronegative versus no *H. pylori* seropositivity.

*H. pylori* seropositivity was not associated with lung cancer in ATBC (Table 2). The OR for any *H. pylori* seropositivity and all lung cancers combined was 1.1 (95% CI: 0.86–1.5). The ORs did not vary by histology (OR: 1.1, 95% CI: 0.75–1.6 for adenocarcinoma and 1.1, 95% CI: 0.77–1.7 for squamous cell carcinoma). In addition, there was no notable variability in the associations for CagA-negative or CagA-positive *H. pylori* seropositivity (Table 2). Results were similar when limited to heavy smokers with more than 35 pack-years (e.g., OR: 0.99, 95% CI: 0.63–1.6; LRT p-value: 0.7 for any *H. pylori* seropositivity and all lung cancers combined).

### Sensitivity analyses

Despite concerns that changes in *H. pylori* seropositivity over time may weaken the association between *H. pylori* seropositivity and cancer risk, associations were similar by median time between baseline serum draw and diagnosis in this study (Table 3). ORs tended to be slightly elevated for cases diagnosed more than 9 years after baseline serum draw, but there was no significant association. In addition, there was little difference in the association between *H. pylori* seropositivity and lung cancer by median age at diagnosis (age 68). For example, the ORs for any *H. pylori* seropositivity and any lung cancer were 1.1 (95% CI: 0.73–1.6) for cases diagnosed at or before age 68 compared to their matched controls and 1.2 (95% CI: 0.79–1.8) for cases diagnosed after age 68 (LRT p-value: 0.7). Similarly, the ORs for CagA-negative *H. pylori* seropositivity and CagA-positive *H. pylori* were 1.0 (95% CI: 0.68–1.6) and 1.1 (95% CI: 0.72–1.7), respectively, for cases diagnosed at or before age 68 and 1.3 (95% CI: 0.83–2.0) and 1.1 (95% CI: 0.69–1.7) for cases diagnosed after age 68 (LRT p-value: 0.7). Results were similar for adenocarcinoma and squamous cell carcinoma separately.

**Table 3:** Association of *Helicobacter pylori* (*H. pylori*) seropositivity with risk of lung adenocarcinoma and squamous cell carcinoma by time to diagnosis in the Alpha-Tocopherol, Beta-Carotene Cancer Prevention Study.

Comparison	Time to diagnosis	OR (95% CI)*		
		Adenocarcinoma	Squamous cell carcinoma	All lung cancer
Any *H. pylori* seropositivity versus none				
	≤9 years	0.95 (0.54–1.7)	1.1 (0.64–1.9)	1.0 (0.70–1.5)
	>9 years	1.3 (0.74–2.2)	1.3 (0.72–2.4)	1.3 (0.86–1.9)
CagA-negative seropositivity versus none				
	≤9 years†	0.96 (0.52–1.8)	1.2 (0.64–2.1)	1.1 (0.70–1.6)
	>9 years‡	1.2 (0.68–2.2)	1.5 (0.79–2.9)	1.4 (0.88–2.1)
CagA-positive seropositivity versus none				
	≤9 years†	0.93 (0.49–1.8)	1.0 (0.57–1.9)	0.99 (0.64–1.5)
	>9 years‡	1.3 (0.72–2.3)	1.0 (0.53–2.1)	1.2 (0.77–1.9)

*Odds ratios (ORs) and 95% confidence intervals (CIs) were adjusted for baseline pack-years and total number of cigarettes per day.

† Derived from a single model for CagA-seropositive and CagA-seronegative versus no *H. pylori* seropositivity for all cases diagnosed ≤9 years after baseline blood draw and their paired controls.

‡ Derived from a single model for CagA-seropositive and CagA-seronegative versus no *H. pylori* seropositivity for all cases diagnosed >9 years after baseline blood draw and their paired controls.

## Discussion

Given our preliminary evidence from existing data in ATBC supporting an inverse association between *H. pylori* seropositivity and lung adenocarcinoma on the one hand, and previous published studies supporting a positive association on the other, we carefully designed a large nested case-control study to thoroughly address the hypothesis that *H. pylori* is associated with lung cancer. In this well-powered nested case-control study, we found no evidence of an association between *H. pylori* and lung cancer. Neither overall *H. pylori* seropositivity nor CagA-specific *H. pylori* seropositivity were associated with lung cancer. Results were equally null for lung adenocarcinoma and squamous cell carcinoma. Sensitivity analyses by time since diagnosis and age had little effect.

The results of previous studies of *H. pylori* seropositivity and lung cancer have been mixed. Of the five previous small case-control studies, three found moderate to strong increased risk of lung cancer associated with *H. pylori* seropositivity [13], [14], [25], and two found only weak, non-statistically significant evidence of a positive association [23], [24]. Two studies provided no information regarding smoking status and lung histology [14], [23], and three provided limited information on smoking status [13], [24], [25]. Only one previous small study of 22 squamous cell carcinoma cases and 21 adenocarcinoma cases provided data on *H. pylori* seropositivity by lung histology; the association in that study did not vary by lung histology [13]. None of these studies evaluated factors like smoking that might affect the association between *H. pylori* seropositivity and lung cancer. Given this lack of adjustment for potential confounders and the fact that small studies are more likely to be affected by chance [45] and less likely to publish null findings [46], it unsurprising that previous, small studies found positive (albeit imprecise) associations.

This study improves on previous studies in a number of ways. First, as a case-control study nested within the ATBC cohort, this study provides the only prospective data on *H. pylori* seropositivity and subsequent risk of lung cancer. With 700 lung cancer cases, this study is nearly 10 times larger than the next largest study and is well powered to assess differences by histology (adenocarcinoma and squamous cell carcinoma). In addition, all participants were smokers at baseline, so there is less variation in smoking habits and therefore less potential for confounding by smoking. As a well-characterized epidemiologic cohort, ATBC has extensive information about potential confounders, which allowed us to carefully control for cumulative smoking exposure and thoroughly assess other potential confounders. Finally, the association between *H. pylori* and gastric cancer has already been established in this population using these same assays, demonstrating the adequacy of these assays.

Although the inclusion of only Finish male smokers is beneficial in limiting the potential for confounding, it also limits the generalizability of these findings. This study could not address the association between *H. pylori* seropositivity and subsequent risk of lung cancer in women. In addition, never smokers may have a different risk of lung cancer associated with *H. pylori* seropositivity than ever smokers. Although unlikely, it is possible that the potential biologic mechanisms described in the Introduction (e.g., systemic inflammation from gastric *H. pylori* infection) may be gender specific or may only be evident in never smokers.

This study provides the most definitive findings for *H. pylori* seropositivity and lung cancer to date. In this population of Finish male smokers, *H. pylori* seropositivity was not associated with lung cancer. Although these findings are not entirely generalizable to other populations (e.g., never smokers), it seems unlikely that *H. pylori* is involved in lung carcinogenesis.
